# Ocular injuries caused by Japanese black bears: a 10-year retrospective analysis of severity and clinical outcomes

**DOI:** 10.3389/fmed.2026.1922412

**Published:** 2026-09-24

**Authors:** Manabu Suzutani, Ryo Mukai

**Affiliations:** Department of Ophthalmology, Fukushima Medical University, Fukushima, Japan

**Keywords:** bear attack, globe rupture, Japanese black bear, ocular trauma, open globe injury, *ursus thibetanus*

## Abstract

**Background:**

Although bear-related injuries are increasing in Japan, ocular trauma associated with Japanese black bear (*Ursus thibetanus japonicus*) attacks has rarely been investigated. We investigated the clinical features and outcomes of bear-related ocular injuries managed at Fukushima Medical University Hospital over the past decade.

**Methods:**

Eight patients who were transported to Fukushima Medical University Hospital for bear-related trauma and subsequently referred to the Department of Ophthalmology for periocular injuries between April 2016 and March 2026 were retrospectively reviewed. Patient demographics, location of injury, the presence of globe rupture, extraocular muscle laceration, eyelid laceration, and facial bone fracture, as well as final best-corrected visual acuity, were evaluated.

**Results:**

Eight patients (mean age 66.6 ± 20.4 years; seven men and one woman) were included. All patients sustained eyelid lacerations. Open globe injury was observed in four eyes (50%), all classified as rupture. Three were surgically confirmed, and one was diagnosed based on clinical and CT findings without surgical exploration. Three of the four globe ruptures (75%) occurred in mountainous areas, and the remaining case in a rural settlement. Half of the cases (4/8) occurred during the most recent two years (2024–2025).

**Conclusion:**

Among bear-related ocular injuries referred to our department over the past 10 years, half involved globe rupture, with the majority occurring in mountainous areas. As bear-related trauma may continue to increase in northern Japan, the establishment of regional management protocols and multidisciplinary collaboration is warranted.

## Introduction

The number of human injuries inflicted by Japanese black bears in Japan has been steadily increasing. According to reports from the Ministry of the Environment, which oversees data on bear-related injuries, the annual number of victims rose from 56 nationwide in 2015 to a record high of 238 in 2025 (1). This rise is attributed to multiple factors, including an increase in the bear population due to a declining number of hunters and the shortage of nuts that constitute their staple food, which has driven bears into urban areas (2). In Japan, this new type of bear that has begun to appear in urban areas is referred to as an “urban bear.” These bears are no longer afraid of humans and have become more likely to engage aggressively when encountered. Cubs that learned the taste of human food alongside their mothers in urban areas several years ago have now matured and, recognizing the town as their own territory, are reappearing in residential areas, further aggravating the situation each year.

Among wild animals in Japan, bears exert particularly powerful physical force and can cause extensive injury. Furthermore, when a bear stands upright, its height aligns with the human head, neck, and face, and injuries tend to be concentrated in the facial region. Indeed, approximately 90% of victims have been reported to sustain facial trauma (3), which is thought to result from the bear striking the victim's head, neck, and face first with the claws of its forelimbs while in an upright posture (4, 5), frequently accompanied by facial bone and skull fractures (3, 6).

Fukushima Prefecture is located in the Tohoku region of northeastern Japan and has a population of approximately 1.7 million (7). It is a predominantly rural area surrounded by extensive forested mountains, where residents occasionally sustain bear-related injuries (8). Herein, we report bear-related injuries treated at our institution over the past 10 years.

## Methods

This retrospective observational case series was conducted at Fukushima Medical University in accordance with the tenets of the Declaration of Helsinki [Institutional Review Board approval number: S2026-02].

Patients who were transported to Fukushima Medical University Hospital (hereafter referred to as “our hospital”) for Japanese black bear-related trauma between April 2016 and March 2026, and who were subsequently referred to our department from the initial treating department (mainly the Department of Emergency Medicine) for periocular injuries, were included in this study. A retrospective review was conducted based on the medical records of the included patients. The variables investigated were patient demographics (age, sex), circumstances of injury (month and place of injury), the affected eye, the presence of open globe injury and concomitant periocular injuries (extraocular muscle laceration, eyelid laceration, and facial bone fracture), the best-corrected visual acuity (BCVA) at presentation and at the final visit to our hospital, duration of follow-up, and final anatomical status (globe retained, phthisis bulbi, or enucleation).

Duration of follow-up was calculated as the interval from the initial ophthalmological consultation to the last documented visit at our institution. Due to the acute nature of these injuries, formal ophthalmological visual acuity testing was not always feasible on initial presentation. In such cases, presenting visual acuity was recorded using bedside estimates obtained in the emergency department: light perception (LP), hand motion (HM), counting fingers (CF), or no light perception (NLP). These qualitative measures were subsequently converted to logMAR equivalents in accordance with the conversion values proposed by Holladay (14) as follows: CF = 2.0, HM = 2.3, LP = 2.7, and NLP = 3.0. All visual acuities are hereafter reported in logMAR units throughout this manuscript.

All patients underwent computed tomography (CT) at initial presentation, along with anterior segment examination in the emergency department. Globe rupture was suspected when CT findings (e.g., distortion of the globe or hemorrhage-filled vitreous cavity) were combined with characteristic clinical findings. In suspected cases, surgical exploration in the operating room was performed for definitive confirmation, unless the patient elected against it.

## Results

Visual acuity data are shown in Table 1. In Case 2, presenting visual acuity could not be assessed due to sedation, but subsequent testing revealed a visual acuity of 0.1 logMAR at the final visit. In Case 4, the eye itself was uninjured and the patient did not report any visual disturbance; therefore, presenting visual acuity was assessed only qualitatively at the emergency department (equivalent to 2.0 logMAR). Subsequent formal testing revealed a visual acuity of 0.5 logMAR, attributable to pre-existing age-related cataract unrelated to the present injury. In Case 5, in whom only eyelid laceration was present without globe involvement and the patient did not report any visual complaint, visual acuity testing was not performed at either presentation or the final visit. In Case 8, visual acuity could not be assessed at either presentation or the final visit due to impaired consciousness. Final anatomical outcomes were globe retention in seven cases and phthisis bulbi in one case (Case 6); no eye underwent enucleation or evisceration.

**Table 1:** List of Japanese black bear-related trauma over the past 10 years at our institution.

	Month	Age/Sex	Place	Affected eye	Open globe (Confirmation method)	Eyelid laceration	Extraocular muscle laceration	Facial fractures	Presenting visual acuity (logMAR)	Final visual acuity (logMAR)	Final anatomical status	Follow—up	Antibiotics
Case 1	August 2016	20s/M	mountainous area	Left	＋ (Surgical exploration)	＋	superior oblique superior rectus	＋	2.7	0.1	Globe retained	111 months	Cefazolin
Case 2	March 2017	60s/M	mountainous area	Right	−	＋	−	−	No data due to sedation	0.1	Globe retained	12 days	Meropenem
Case 3	March 2019	60s/M	mountainous area	Right	＋ (Surgical exploration)	＋	superior oblique lateral rectus	＋	3.0	2.3	Globe retained	62 months	Ampicillin, Sulbactam
Case 4	September 2023	90s/M	field	Right	−	＋	lateral rectus inferior rectus	−	2.0	0.5	Globe retained	2 days	Cefazolin
Case 5	June 2024	70s/M	village	Left	−	＋	−	−	Not tested (no visual complaint)	Not tested (no visual complaint)	Globe retained	2 days	Ampicillin, Sulbactam
Case 6	July 2024	60s/M	mountainous area	Right	＋ (Clinical + CT findings)^*^	＋	unidentifiable due to severe tissue damage	＋	3.0	3.0	Phthisis bulbi	7 months	Ampicillin, Sulbactam
Case 7	October 2025	60s/M	mountainous area	Left	−	＋	unidentifiable due to severe tissue damage	＋	2.0	2.0	Globe retained	7 days	Cefazolin
Case 8	October 2025	80s/F	village	Right	＋ (Surgical exploration)	＋	lateral rectus superior rectus	＋	Not tested (impaired consciousness)	Not tested (impaired consciousness)	Globe retained	26 days	Ampicillin, Sulbactam

Ages are shown by decade.

Presenting and final visual acuities are shown in logMAR units. For qualitative measures obtained at the bedside, the following conversion values were used: CF = 2.0, HM = 2.3, LP = 2.7, and NLP = 3.0 (14).

Follow-up indicates the duration from the initial visit at our hospital until referral to a local clinic.

*Case 6: Globe rupture was diagnosed based on clinical (irregular elevation of the conjunctiva) and imaging (CT showing hemorrhage-filled vitreous cavity) findings, without surgical confirmation, as the patient elected to prioritize facial reconstruction over ophthalmic surgical exploration.

Open globe injury was identified in four cases, all of which were classified as rupture. Of the four cases of globe rupture, three (Cases 1, 3, and 8) were surgically confirmed during ophthalmic exploration in the operating room, whereas one (Case 6) was diagnosed with globe rupture based on clinical and CT findings (vitreous hemorrhage); however, no ophthalmic intervention was performed because the patient elected to prioritize facial reconstruction over ophthalmic surgical exploration. Three of these four cases (75%) occurred in mountainous areas (two during leisure activities and one during work), whereas the remaining case occurred in front of the patient's home in a rural settlement located near the boundary between urban and mountainous areas.

The number of victims within Fukushima Prefecture (including those transported to other hospitals) and the number of cases referred to our department during the same period are shown in Table 2. According to reports from the Ministry of the Environment (data for 2016–2020 were not publicly available) (1), the number of Japanese black bear sightings in Fukushima Prefecture increased from 303 in 2021 to 385 in 2022, 709 in 2023, 618 in 2024, and 1,996 in 2025, accompanied by a modest but concurrent increase in the number of ophthalmology referrals (0, 0, 1, 2, and 2 cases, respectively).

**Table 2:** Annual numbers of bear sightings, human injuries, and ophthalmology referrals in fukushima prefecture (2016–2025).

Year	No. of bear sightings (*n*)	No. of injuries (*n*)	Referrals to ophthalmology (*n*)
2016	—	7	1
2017	—	9	1
2018	—	1	0
2019	—	10	1
2020	—	9	0
2021	303	3	0
2022	385	7	0
2023	709	15	1
2024	618	6	2
2025	1,996	24	2

Annual numbers of Japanese black bear (*Ursus thibetanus japonicus*) sightings, bear-related human injuries, and referrals to our ophthalmology department in Fukushima Prefecture from 2016 to 2025. Data on sightings from 2016 to 2020 were not publicly available.

Case 1 and Case 3 have been previously reported in a Japanese-language publication from our institution (9); however, that report has limited accessibility outside Japan because it was written in Japanese. Because both cases involved globe rupture—the most severe form of ocular trauma in this cohort—we considered it valuable to include these two cases within a comprehensive 10-year single-center series, which strengthens the systematic characterization of bear-related ocular injuries and may provide useful guidance for future clinical practice.

Case 1 (Figure 1). The patient was referred to our department because of an eyelid laceration. The anterior chamber depth was preserved with no hyphema; however, circumferential subconjunctival hemorrhage was observed, and prolapse of the superior oblique muscle through the eyelid laceration was noted. Computed tomography (CT) demonstrated enophthalmos, and surgery was indicated because of suspected globe rupture. Upon incision of the superior conjunctiva, a transverse scleral laceration was found beneath the insertion of the superior rectus, with vitreous and choroidal prolapse, which were debrided and closed with 8-0 nylon sutures. Vitreous hemorrhage was present on funduscopy, but no retinal detachment was observed, and the surgery was terminated. Considering the high risk of retinal detachment caused by traction from the incarcerated vitreous, cataract surgery and pars plana vitrectomy were performed on postoperative day 12 to remove the vitreous hemorrhage and release the vitreous incarceration at the scleral laceration. No retinal detachment was observed; endolaser photocoagulation was applied around the scleral laceration, gas tamponade was performed, and the surgery was concluded. Although cyclotropia persisted at the final visit, the patient did not report diplopia due to persistent ptosis.

**Figure 1:**
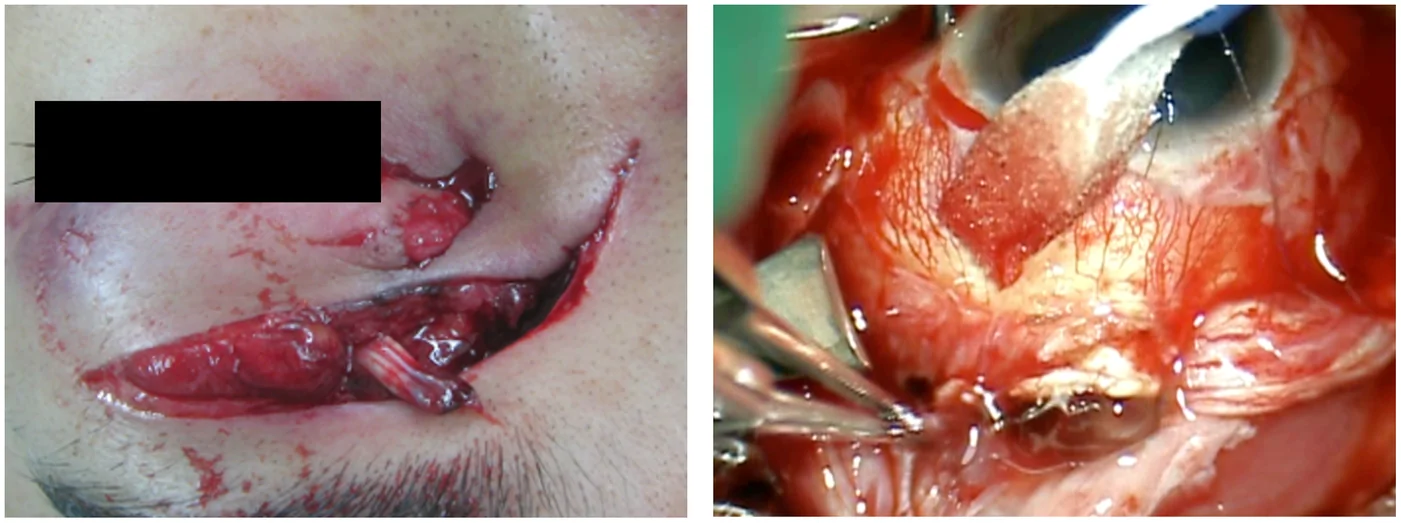
Eyelid laceration and globe rupture in case 1. The muscle exposed through the eyelid laceration was identified as the superior oblique. The site of the eyelid laceration did not coincide with that of the globe rupture, and no penetrating wound was observed; these findings suggest that the globe rupture was caused by high-energy blunt trauma rather than direct claw penetration. Prolapse of the vitreous and choroid was noted through the rupture site.

Case 3 (Figure 2). Claw-induced lacerations were observed across the entire face. Hyphema and vitreous hemorrhage were present, and the fundus could not be visualized. Although circumferential subconjunctival hemorrhage was noted, no scleral rupture site was identified from the ocular surface, and the globe contour was preserved. Under general anesthesia, conjunctival incision revealed that the superior oblique and lateral rectus muscles were transected at their insertions, and a scleral laceration was identified extending circumferentially over 180°, from the posterior aspect of the inferior margin of the lateral rectus insertion to the nasal side of the superior rectus. Tissue suspected to be choroid or retina had prolapsed; non-reducible portions were excised, and the sclera was closed with 8-0 nylon sutures. Although the superior oblique muscle was severely damaged and could not be reattached, the lateral rectus and the superior rectus—which had been detached for surgical exposure—were sutured back to the eye wall, and the surgery was concluded. On postoperative day 7, ultrasonography revealed possible retinal detachment with proliferative tissue, and reoperation was performed. The entire retina had become incarcerated in the previous rupture site, resulting in a funnel-shaped detachment. As release of the incarceration was difficult, a circumferential retinectomy was performed, the subretinal proliferative tissue was peeled, circumferential endolaser photocoagulation was applied, and gas tamponade was performed. Although the retina was reattached under gas tamponade, retinal detachment recurred on postoperative day 61 following gas absorption. On postoperative day 65, pars plana vitrectomy combined with scleral-fixated intraocular lens implantation was performed. The retina was circumferentially detached with marked proliferative changes; the retina was reattached using perfluorocarbon liquid, photocoagulation was applied to the entire retina except for the macula, and silicone oil tamponade was performed. Subsequently, fundus evaluation became impossible because of corneal opacification.

**Figure 2:**
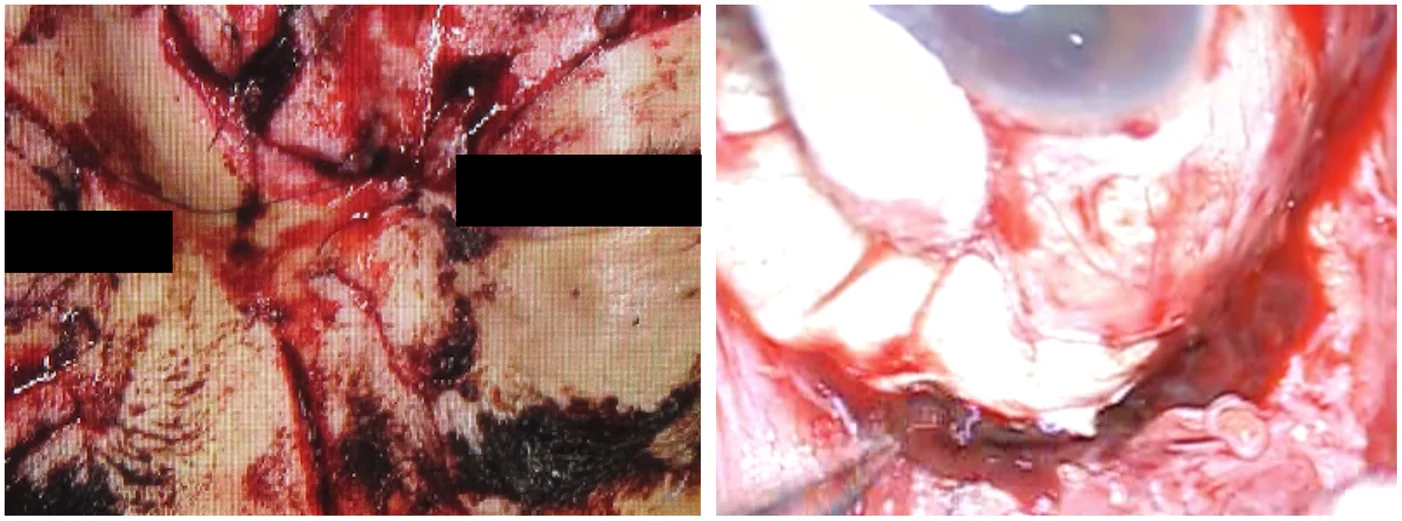
Facial lacerations and globe rupture in case 3. Numerous claw-induced lacerations are seen on the face. The globe rupture was located at the superior equator, through which the vitreous and tissue suspected to be the choroid or retina had prolapsed.

Case 6 (Figure 3). Hyphema was present and globe rupture was suspected; however, because the affected eye had a visual acuity of 3.0 logMAR and the patient elected to prioritize facial reconstruction over ophthalmic surgical exploration, no ophthalmic intervention was performed. The diagnosis of globe rupture was based on (1) irregular elevation of the conjunctiva and (2) CT findings demonstrating that the vitreous cavity was entirely filled with blood, without surgical confirmation. The affected eye subsequently developed phthisis bulbi.

**Figure 3:**
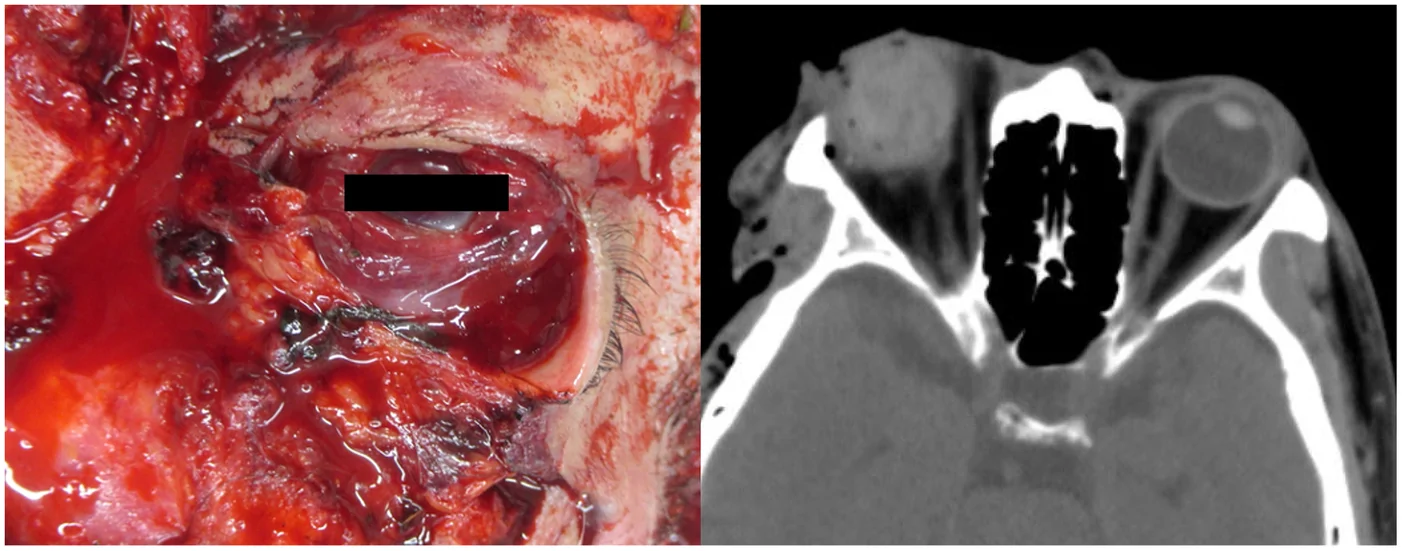
Periocular photograph and computed tomography (CT) of case 6. Left: The soft tissue extending from the glabella to the right upper eyelid had been avulsed. Hyphema was present, and irregular elevation of the conjunctiva was noted, raising suspicion of globe rupture. Right: Axial CT image showing the right vitreous cavity entirely filled with blood, further supporting the suspicion of globe rupture. Because the affected eye had a visual acuity of 3.0 logMAR and the patient elected to prioritize facial reconstruction over ophthalmic surgical exploration, no ophthalmic intervention was performed; the diagnosis of globe rupture was therefore based on clinical and imaging findings without surgical confirmation.

Case 8 (Figure 4). Circumferential subconjunctival hemorrhage was observed. When the globe was grasped with toothed forceps, vitreous prolapse was noted from the superior sclera, and surgery was performed. The rupture site originated from a previous cataract surgical wound, and intraocular lens dislocation was also observed; closure was performed using 7-0 Vicryl sutures. Because vitreous hemorrhage was also present, pars plana vitrectomy was performed for irrigation; however, the fundus could not be visualized, and the surgery was terminated. Although reoperation was considered later, it was not performed because the patient's consciousness did not recover.

**Figure 4:**
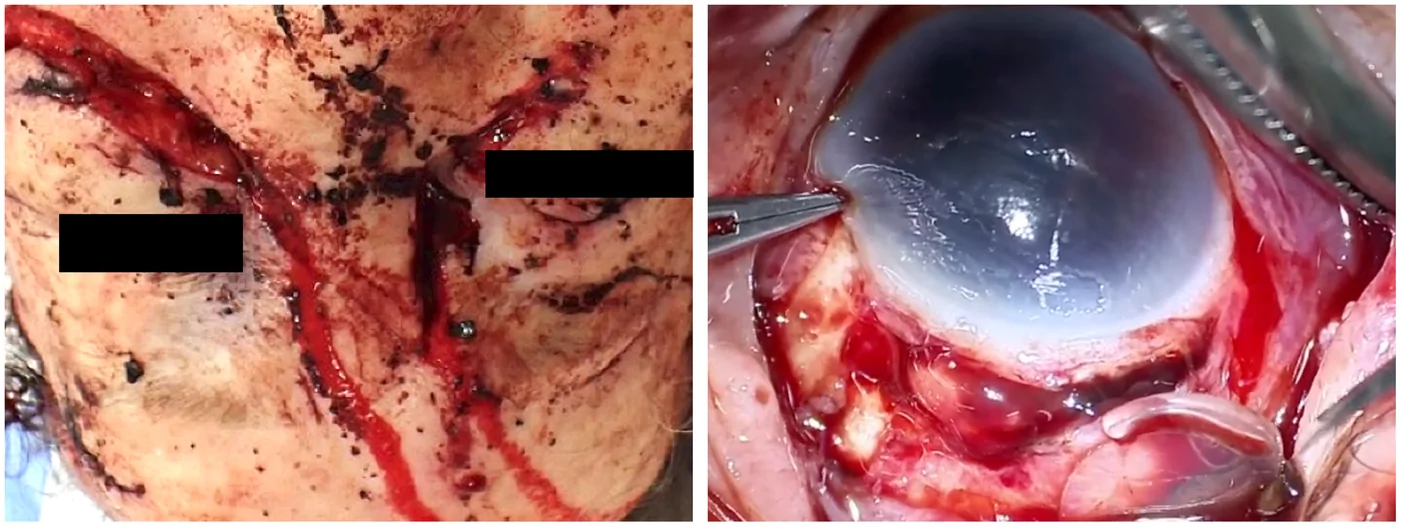
Facial laceration and globe rupture in case 8. Although facial lacerations were more severe on the left side, globe rupture was identified in the right eye. The rupture originated from a previous cataract surgical wound, with prolapse of the intraocular lens and vitreous.

Antibiotics and tetanus toxoid were administered by the Department of Emergency Medicine in all cases. In our department, topical moxifloxacin hydrochloride was prescribed for patients with periorbital wounds. No systemic or local infections occurred.

## Discussion

Bear-related trauma is a complex high-energy injury comprising both sharp injuries caused by the claws and blunt injuries caused by powerful impact. When a bear stands upright and swings its forelimbs downward, the claws produce multiple parallel lacerations on the victim's head, neck, and face, while the underlying impact energy concurrently produces a wide range of blunt injuries, including facial bone fractures, skull fractures, and globe rupture. To our knowledge, this is the first English-language systematic report of bear-related ocular injuries focusing specifically on the ophthalmological perspective, based on a 10-year single-center experience in a region with a high prevalence of human-bear encounters.

Regarding the appearance of Japanese black bears in human habitations in Japan, poor crops of beech and Mongolian oak, which are the main food sources in autumn, have been reported to be associated with increased sightings and nuisance captures. However, the recent rise in sightings cannot be explained by nut crop conditions alone, and meteorological factors, changes in the satoyama environment, depopulation of mountainous areas, and the presence of anthropogenic attractants such as crops and food waste may be involved in a complex manner (10, 11). In any case, the distance between bears and humans has become shorter than before, and concerns regarding an increased incidence of injury are expected to grow. Indeed, although only eight patients were referred to our department for periocular trauma over this 10-year period, four cases (50% of the total) occurred during the most recent two years from 2024 onward. In 2025 alone, of the 24 patients reported to have sustained bear-related trauma in Fukushima Prefecture, four were transported to our hospital for multidisciplinary treatment, and two of these had ocular trauma. Although Tsuchida et al. reported that approximately 90% of victims sustained facial trauma (3), minor facial lacerations managed at neighboring institutions were not included in the present study, and the precise rate of facial trauma across the entire prefecture could not be verified. On the other hand, because cases requiring ophthalmic surgical intervention—such as globe rupture—are centralized at our hospital within the prefecture, the ophthalmology referral rate of 8.8% demonstrated in this study (8 referred cases/91 total bear-related victims in Fukushima Prefecture over the past 10 years) may serve as an indicator of the incidence of ocular trauma caused by Japanese black bears. When limited to globe rupture, our rate was lower than the 15% (3/20 cases) reported by Tsuchida et al. (3) and the 16% (8/50 cases) reported by Takahashi et al. (12), both also from university hospitals; however, because bear-related injuries are sustained from natural-disaster-like encounters with a wild animal, the circumstances of injury, transport background, and the selection process by which cases are referred to ophthalmology differ, making direct comparisons difficult.

Open globe injury is classified as rupture, in which a blunt force produces a full-thickness wound of the eye wall, or laceration, in which a sharp object produces a full-thickness cut of the eye wall (13). In all four cases of globe rupture in the present study, although sharp lacerations corresponding to claw marks were observed on the face and eyelids, no claw-induced penetrating wounds were observed deeper than the eyelids, and the appearance of the corneoscleral laceration was also consistent with rupture. A possible mechanism of globe rupture is that the powerful impact energy from the bear's forelimb was applied to the periorbital region from the inferior or temporal side, where the bone is thinner and more vulnerable to external force than the superior or nasal side, and was then indirectly transmitted to the globe through the eyelid soft tissue or the orbital wall. In other words, a contrecoup-like mechanism, in which the injury occurs not at the site of impact but at the opposite or a distant site, may have been involved. Furthermore, fear-induced eyelid closure and upward rotation of the globe due to the Bell's phenomenon are presumed to have been present at the time of injury. As a result, when energy was applied from the front or side of the face, stress was concentrated through this contrecoup-like mechanism at the thin superior sclera near the muscle insertions or the equator, leading to globe rupture. In addition, three of the four globe ruptures (75%) in the present study occurred in mountainous areas. As a basis for interpreting this geographical distribution of injury locations, the concept of the ideal despotic distribution proposed by Fretwell et al. may be relevant (15). According to this concept, dominant individuals occupy high-quality habitats and force subordinate individuals into lower-quality ones, resulting in a spatial segregation of the population. Dominant individuals occupying high-quality habitats in mountainous areas are larger and more experienced and may exhibit persistent attacks when encountered within their territories, whereas individuals that appear near human settlements tend to be subordinate or subadult and may exhibit only defensive or incidental attacks, resulting in relatively lower severity. The finding that globe rupture in the present study predominantly occurred in mountainous areas may be consistent with this hypothesis, and suggests that clinicians should maintain a particularly high index of suspicion for severe ocular trauma, including occult globe rupture, when patients are injured by bears in mountainous environments.

Bears harbor a diverse bacterial flora on their claws and in their oral cavity; therefore, wounds are heavily contaminated and carry a high risk of polymicrobial infection (3, 6). Bacteria isolated from the oral cavity and bite wounds of bears include aerobic species such as *Streptococcus*, *Staphylococcus*, *Escherichia coli*, and *Enterobacter*, together with anaerobic species, demonstrating polymicrobial infection (16); in particular, *Serratia*, *Aeromonas hydrophila*, *Bacillus cereus*, and *Enterococcus* species have been isolated from a grizzly bear bite wound (17). At our hospital, antibiotics were selected by the Department of Emergency Medicine based on initial assessment of the severity and degree of contamination of each individual case; no wound infections occurred, and all patients had a favorable clinical course. Multidisciplinary initial management is important in bear-related trauma, and the development of in-hospital protocols remains a future challenge. Although post-exposure prophylaxis for rabies is not required for bear-related injuries occurring within Japan because the country is a rabies-free area, this does not apply to injuries sustained in regions where bears can serve as reservoirs of the rabies virus, such as North America, Eastern Europe, and Southeast Asia (16).

This study has several limitations. First, it is a single-center retrospective observational study with a limited sample size of eight cases. Second, cases that did not require ophthalmology referral despite facial lacerations, and mild cases managed at other institutions, were not included; therefore, referral bias cannot be completely excluded. Third, the sighting data for Fukushima Prefecture have been compiled only since fiscal year 2021, and direct comparison with sighting trends before that period is not possible. Further accumulation of cases is needed to better characterize the clinical features and prognostic factors of ocular injuries in Japanese black bear-related trauma. Fourth, presenting visual acuities were originally assessed qualitatively at the bedside and subsequently converted to logMAR equivalents based on standard conversion values (14); because these logMAR equivalents are approximations rather than direct measurements, comparison with logMAR-measured final visual acuity should be interpreted with caution.

## Conclusion

In the present study, we reported the clinical characteristics of ocular injuries caused by Japanese black bears in Fukushima Prefecture over the past 10 years. As the severity and clinical course varied substantially among cases, the findings cannot be uniformly generalized; nevertheless, the best possible management should be provided according to the circumstances of each case. Given the potential for occult globe rupture and severe visual impairment, a low threshold for urgent computed tomography and ophthalmological consultation should be considered whenever feasible in cases of periocular laceration caused by a bear. Furthermore, as bear-related trauma may continue to increase in the Tohoku region, including Fukushima Prefecture, the establishment of regional initial-management protocols and multicenter accumulation of cases are warranted.

## Data Availability

The original contributions presented in the study are included in the article/Supplementary Material, further inquiries can be directed to the corresponding author/s.
